# Supplementary material for: Conformational transitions and allosteric modulation in a heteromeric glycine receptor
Source: Nat Commun. 2023 Mar 13;14:1363. doi: 10.1038/s41467-023-37106-7 (PMC10011588; doi:10.1038/s41467-023-37106-7)
Supplement: Supplementary file 1 — Supplementary Information [file 41467_2023_37106_MOESM1_ESM.pdf]

Supplementary Information

Conformational transitions in a heteromeric glycine receptor associated with antagonism, agonism, and positive allosteric modulation

Gibbs et al

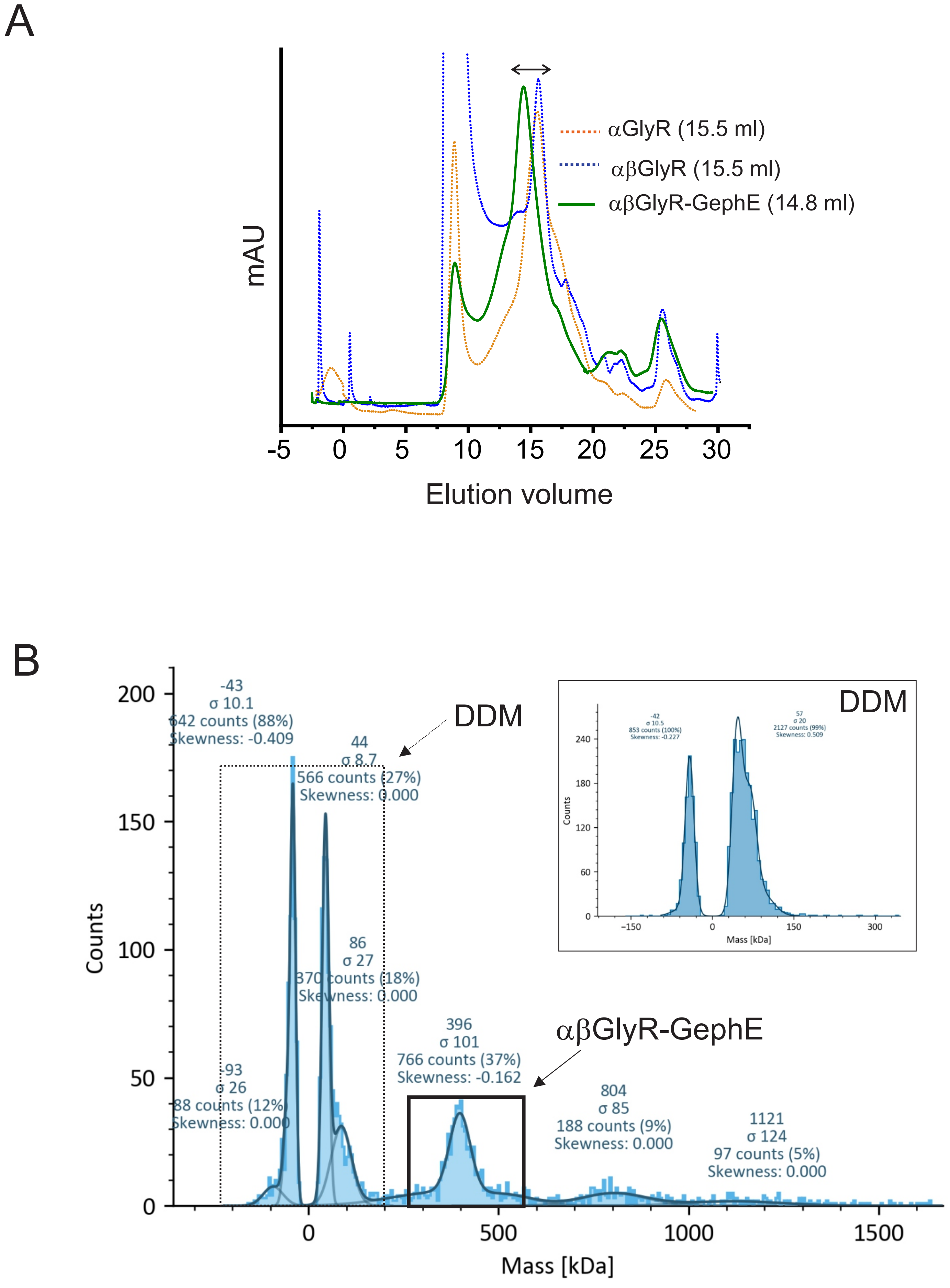
A
αGlyR (15.5 ml)
αβGlyR (15.5 ml)
αβGlyR-GephE (14.8 ml)
mAU
-5
0
5
10
15
20
25
30
Elution volume
B
-43
σ 10.1
642 counts (88%)
Skewness: -0.409
44
σ 8.7
566 counts (27%)
Skewness: 0.000
DDM
86
σ 27
370 counts (18%)
Skewness: 0.000
-93
σ 26
88 counts (12%)
Skewness: 0.000
396
σ 101
766 counts (37%)
Skewness: -0.162
αβGlyR-GephE
804
σ 85
188 counts (9%)
Skewness: 0.000
1121
σ 124
97 counts (5%)
Skewness: 0.000
Counts
0
50
100
150
200
0
500
1000
1500
Mass [kDa]
DDM
-42
σ 10.5
853 counts (100%)
Skewness: -0.227
57
σ 20
2127 counts (99%)
Skewness: 0.509
Counts
0
60
120
180
240
-150
0
150
300
Mass [kDa]

Supplementary Figure 1: Biochemical analysis of GlyRs. A) Gel filtration profiles of purified α1GlyR, α1βGlyR, α1βGlyR (coexpressed with Geph-E). The peak corresponding to the pentamers is left-shifted for α1βGlyR (coexpressed with Geph-E) and western blot analysis showed the peak contained all three expressed proteins. B) Mass Photometry (MP) distribution analysis of the α1βGlyR-Geph-E sample. *Inset* shows the analysis for the sample buffer. The measured mass of 396 kDa is close to the expected mass of 405.5 kDa.

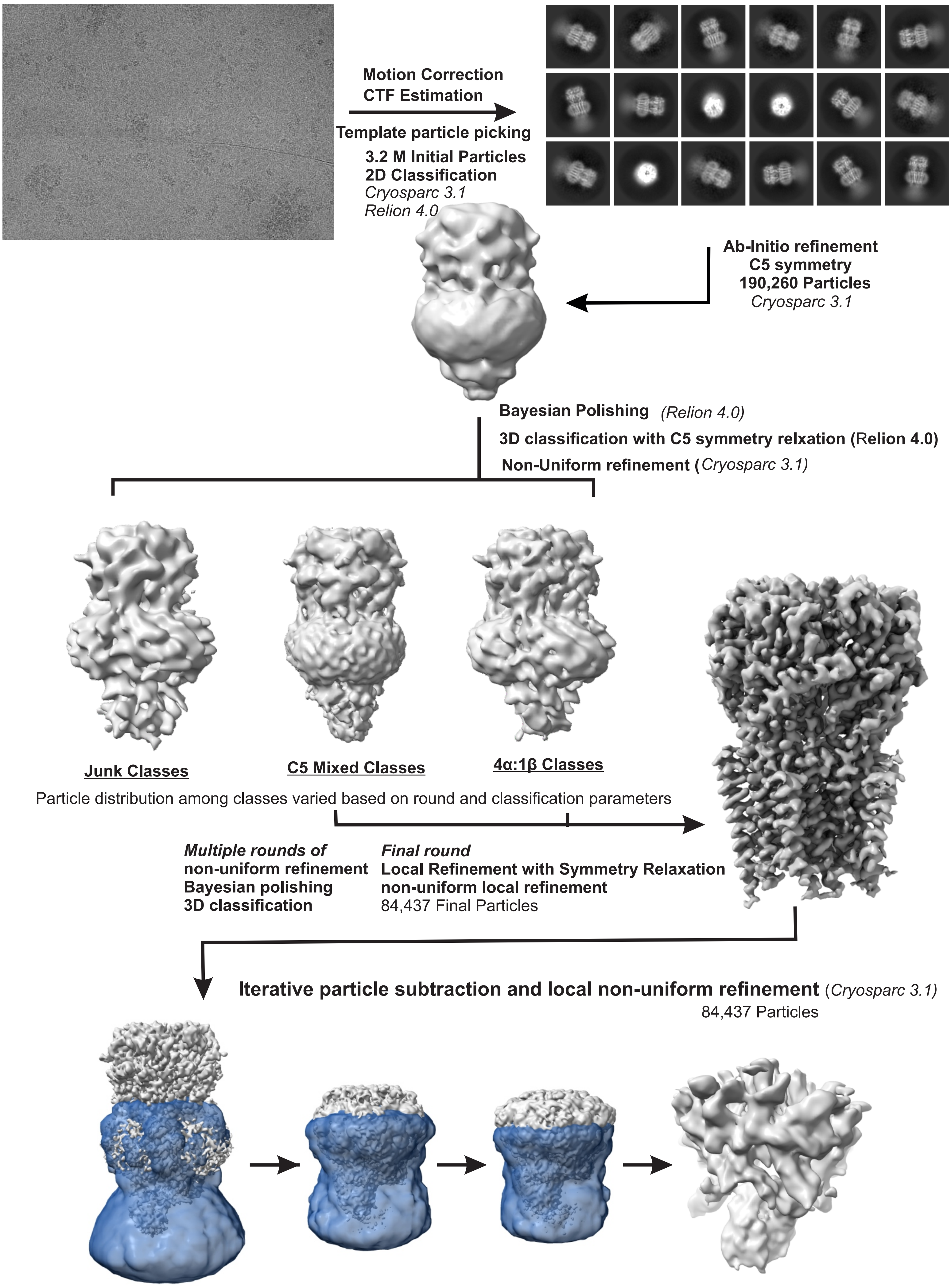
Motion Correction
CTF Estimation
Template particle picking
3.2 M Initial Particles
2D Classification
Cryosparc 3.1
Relion 4.0
Ab-Initio refinement
C5 symmetry
190,260 Particles
Cryosparc 3.1
Bayesian Polishing (Relion 4.0)
3D classification with C5 symmetry relxation (Relion 4.0)
Non-Uniform refinement (Cryosparc 3.1)
Junk Classes
C5 Mixed Classes
4α:1β Classes
Particle distribution among classes varied based on round and classification parameters
Multiple rounds of
non-uniform refinement
Bayesian polishing
3D classification
Final round
Local Refinement with Symmetry Relaxation
non-uniform local refinement
84,437 Final Particles
Iterative particle subtraction and local non-uniform refinement (Cryosparc 3.1)
84,437 Particles

Supplementary Figure 2: Flow chart description of data processing of αβ-GlyR. Images shown are from αβGlyR-Stry data processing. Full details are described in the Methods section. All datasets were refined to a single conformation.

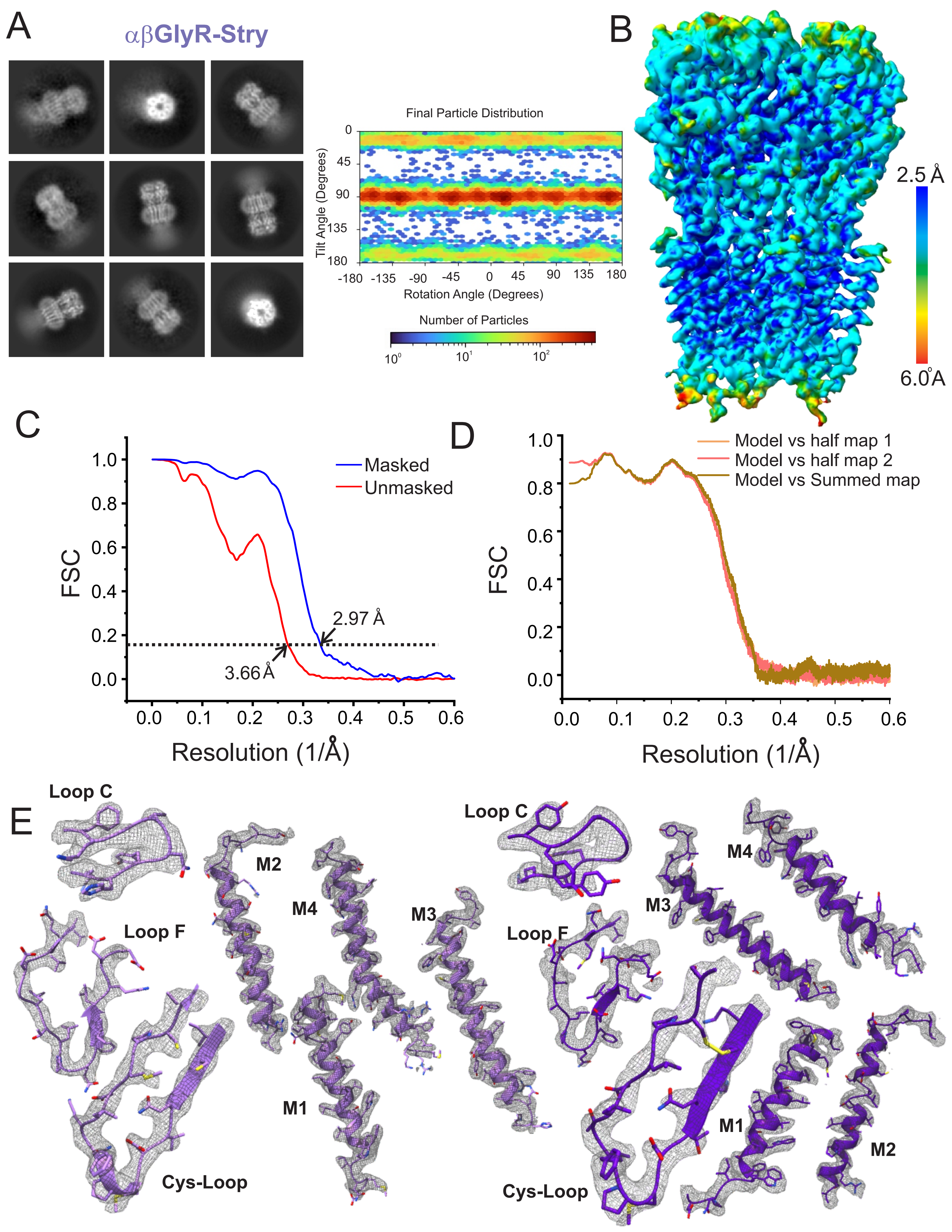

A
αβGlyR-Stry
Final Particle Distribution
Tilt Angle (Degrees)
Rotation Angle (Degrees)
Number of Particles
B
2.5 Å
6.0 Å
C
FSC
Masked
Unmasked
2.97 Å
3.66 Å
Resolution (1/Å)
D
Model vs half map 1
Model vs half map 2
Model vs Summed map
FSC
Resolution (1/Å)
E
Loop C
Loop F
Cys-Loop
M1
M2
M3
M4

Supplementary Figure 3: Map/Model validations for αβGlyR-Stry data. A) Representative 2D classes and final particle distributions of αβGlyR-Stry particles done in cryoSPARC (3.3.1). B) Local resolution map as determined by ResMap. C) FSC curve calculated using RELION 4.0 Post-Processing D) and Map/Model correlations calculated using PHENIX routine mtriage. E) Corresponding map and model density shown for the αB subunit (light purple) and β subunit (dark purple). Map thresholds are set at the following levels αB: M1-M4 (0.16), Loop C, Cys Loop (0.26), Loop F (0.24) β: M1-M4 (0.16). β: Loop C and Cys Loop (0.26) and Loop F (0.24).

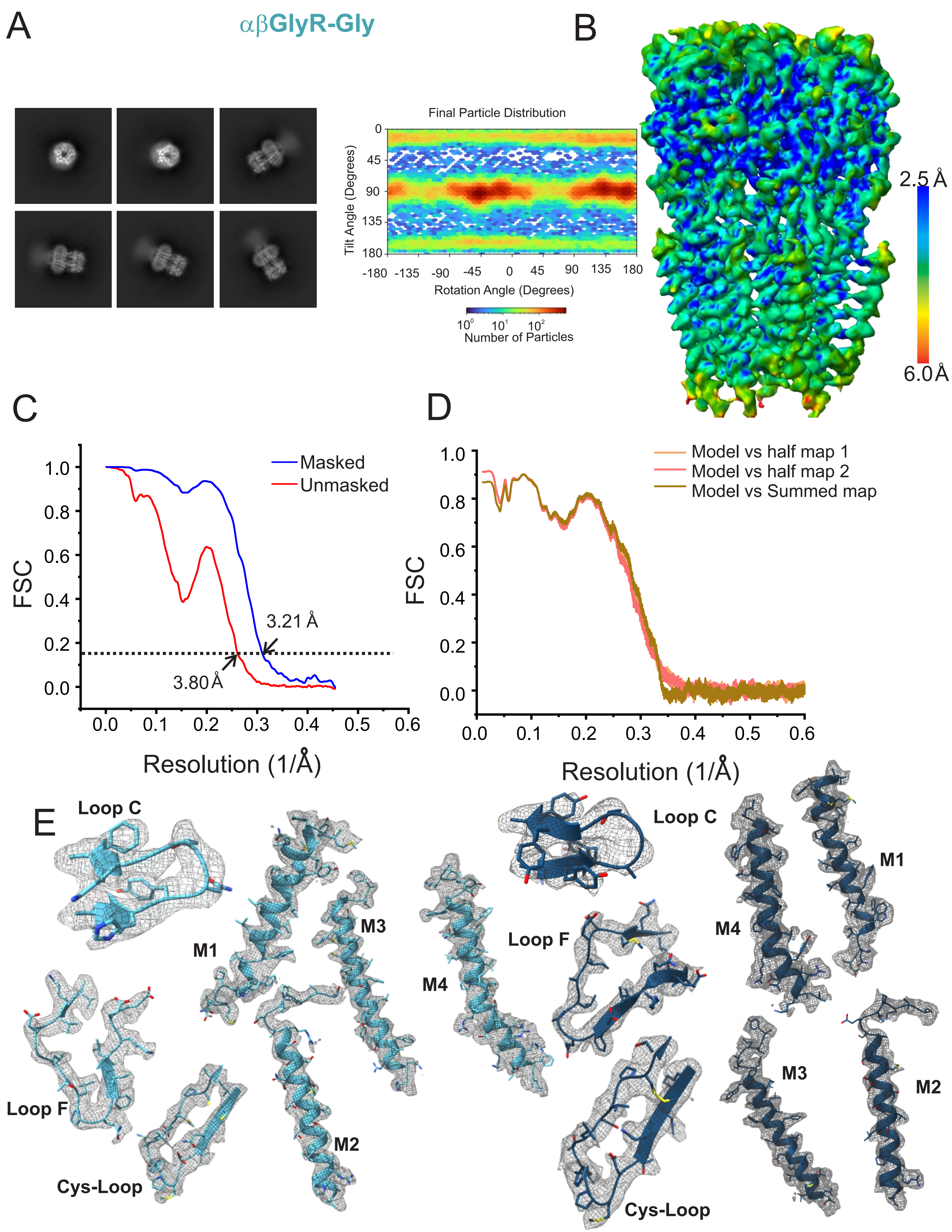
A
αβGlyR-Gly
Final Particle Distribution
Tilt Angle (Degrees)
Rotation Angle (Degrees)
Number of Particles
B
2.5 Å
6.0 Å
C
FSC
Masked
Unmasked
3.21 Å
3.80 Å
Resolution (1/Å)
D
Model vs half map 1
Model vs half map 2
Model vs Summed map
FSC
Resolution (1/Å)
E
Loop C
M1
M3
M4
Loop F
M2
Cys-Loop

Supplementary Figure 4: Map/Model validations for αβGlyR-Gly data. A) Representative 2D classes and final particle distributions of αβGlyR-Gly particles done in cryoSPARC (3.3.1). B) Local resolution map as determined by ResMap. C) FSC curve calculated by post-processing in Relion 4.0. D) Map/Model correlations calculated using PHENIX routine mtriage. E) Corresponding map and model density shown for the αB subunit (light blue) and β subunit (dark blue). Map thresholds are set at the following levels αB: M1-M4 (0.3), Loop C (0.54), Loop F (0.36), Cys Loop (0.39). β: M1-M4 (0.26), Loop C (0.45), Loop F (0.34) and Cys Loop (0.29).

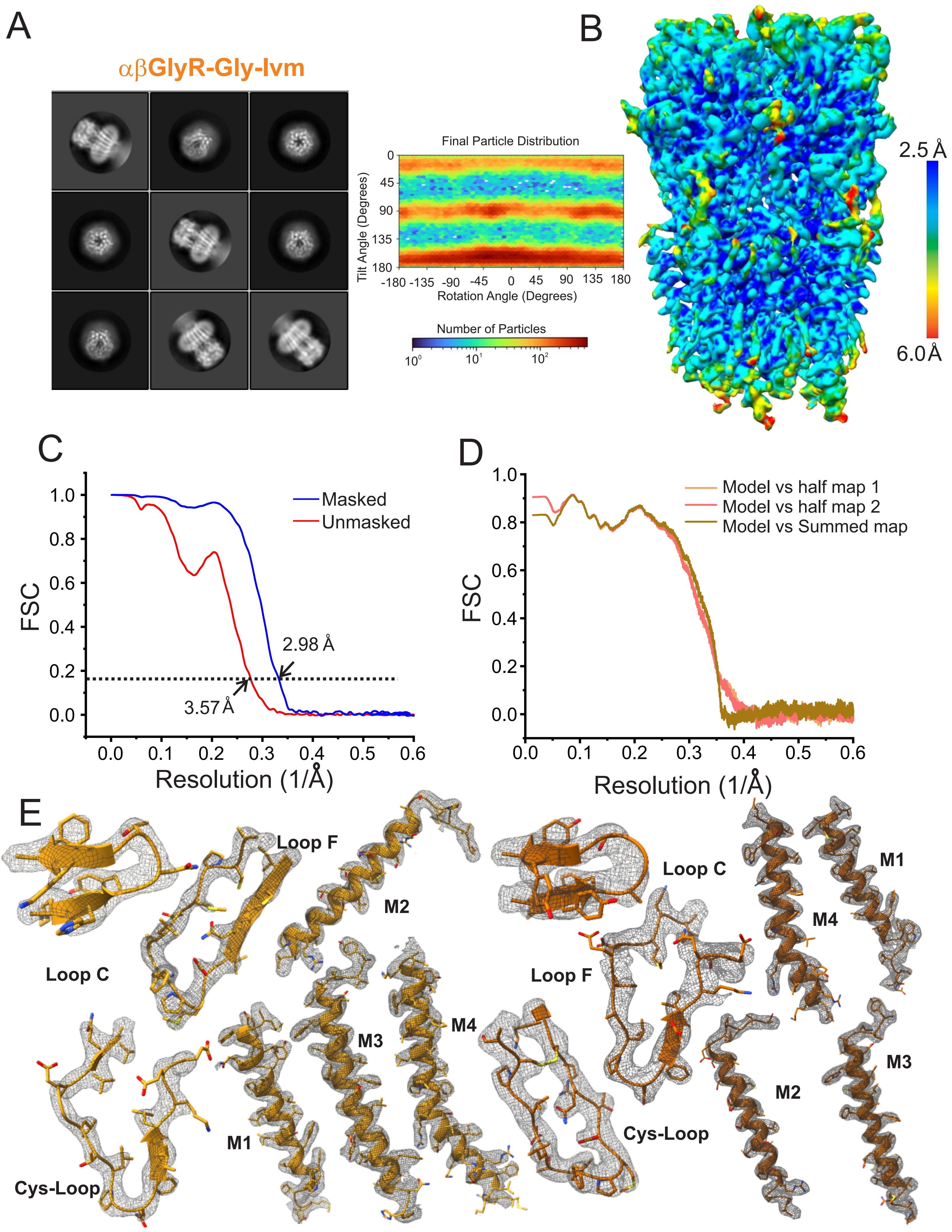

A
αβGlyR-Gly-Ivm
Final Particle Distribution
Tilt Angle (Degrees)
Rotation Angle (Degrees)
Number of Particles
B
2.5 Å
6.0 Å
C
FSC
Masked
Unmasked
2.98 Å
3.57 Å
Resolution (1/Å)
D
FSC
Model vs half map 1
Model vs half map 2
Model vs Summed map
Resolution (1/Å)
E
Loop C
Loop F
M2
M1
M3
M4
Cys-Loop

Supplementary Figure 5: Map/Model validations for αβGlyR-Gly-Ivm data. A) Representative 2D classes and final particle distributions of αβGlyR-Gly-Ivm particles done in Relion (4.0). B) Local resolution map as determined by ResMap. C) FSC curve calculated by post-processing in Relion 4.0. D) Map/Model correlations calculated using PHENIX routine mtriage. E) Corresponding map and model density shown for the αB subunit (yellow) and β subunit (orange). Map thresholds are set at the following levels αB: M1-M4 (0.15), Loop C (0.22), Loop F (0.2), Cys Loop (0.22). β: M1-M4 (0.15), Loop C (0.18), Loop F (0.15), and Cys Loop (0.15).

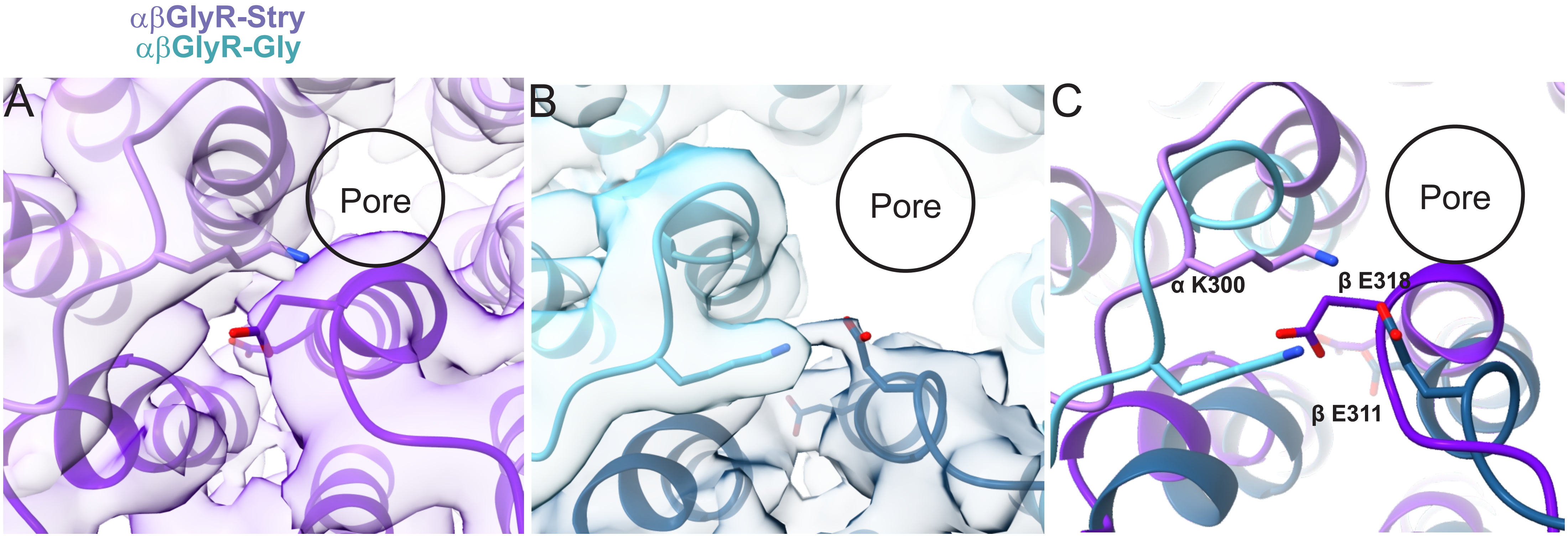
αβGlyR-Stry
αβGlyR-Gly
A
Pore
B
Pore
C
Pore
α K300
β E318
β E311

Supplementary Figure 6: Conformational transitions of βGlyR-specific glutamates near the channel pore. A) B) Map/model alignments for the E318, E311 and K300 for αβGlyR-Stry (σ=0.07) and αβGlyR-Gly (σ=0.11), respectively. C) Model comparison of β-specific glutamates in αβGlyR-Stry (purple) and αβGlyR-Gly (blue) models. The βGlyR subunit is shown in a darker shade.

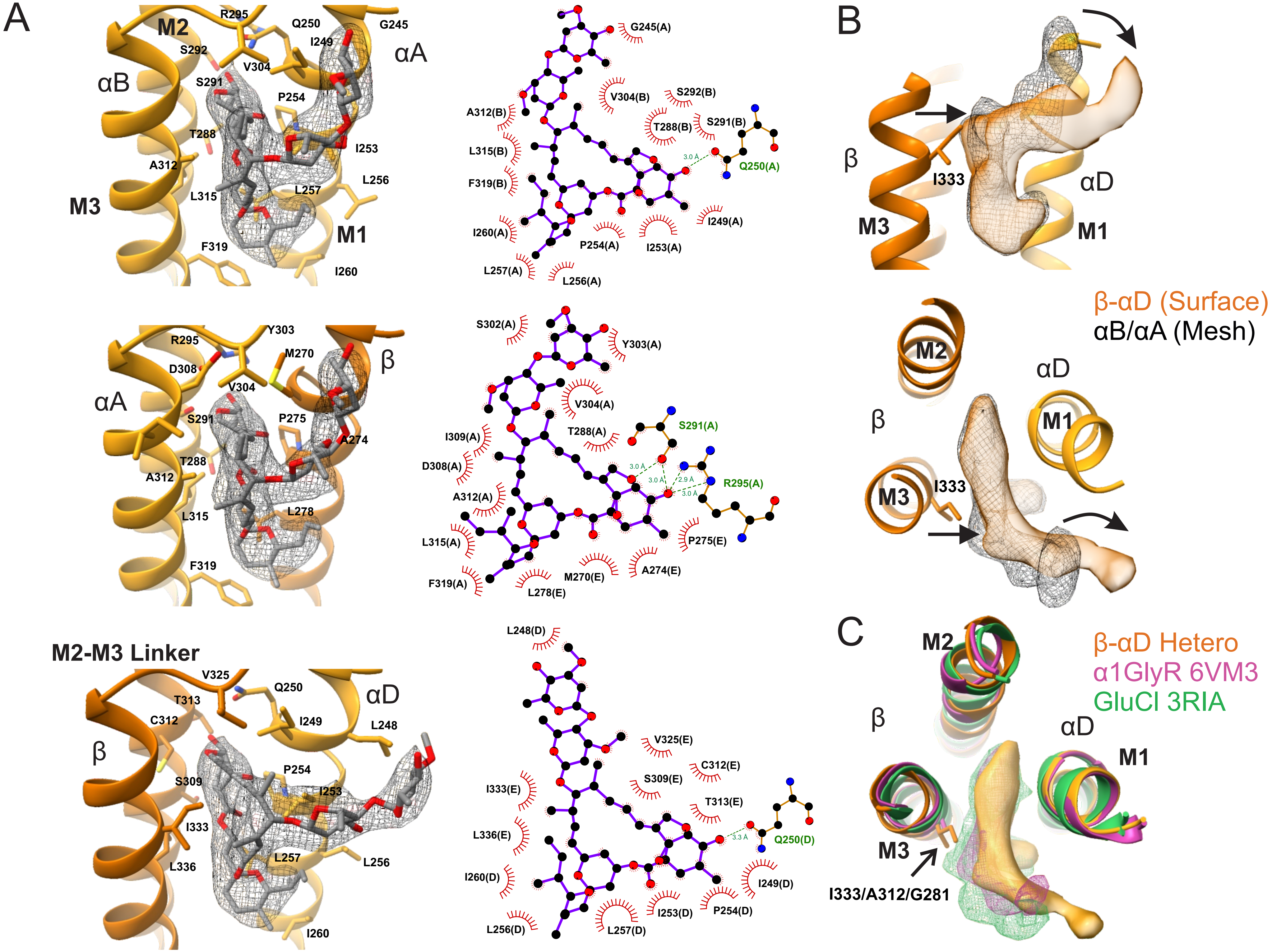
A
M2
R295
Q250
G245
I249
αA
S292
αB
V304
S291
P254
T288
I253
A312
L256
M3
L315
L257
M1
F319
I260
G245(A)
V304(B)
S292(B)
A312(B)
T288(B)
S291(B)
L315(B)
3.0 Å
Q250(A)
F319(B)
I260(A)
I249(A)
P254(A)
I253(A)
L257(A)
L256(A)
Y303
R295
M270
β
D308
V304
αA
S291
P275
A274
T288
A312
L278
L315
F319
S302(A)
Y303(A)
V304(A)
S291(A)
I309(A)
T288(A)
D308(A)
3.0 Å
2.9 Å
R295(A)
A312(A)
L315(A)
P275(E)
A274(E)
M270(E)
F319(A)
L278(E)
M2-M3 Linker
V325
Q250
αD
T313
C312
I249
L248
β
P254
S309
I253
I333
L257
L256
L336
I260
L248(D)
V325(E)
C312(E)
S309(E)
I333(E)
T313(E)
L336(E)
3.3 Å
Q250(D)
I260(D)
I249(D)
P254(D)
I253(D)
L256(D)
L257(D)
B
β
I333
αD
M3
M1
β-αD (Surface)
αB/αA (Mesh)
M2
αD
β
M1
M3
I333
C
M2
β-αD Hetero
α1GlyR 6VM3
GluCl 3RIA
β
αD
M1
M3
I333/A312/G281

Supplementary Figure 7: Ivermectin interactions and pose at different subunit interfaces. A) The full set of ivermectin interactions and LigPlot analysis. Map density is shown at σ=0.11. The αB/αA, αA/β and β/αD interfaces are respectively shown in the top, middle and bottom rows. B) Comparison of the ivermectin density at the αB/αA (mesh), αA/β, β/αD (transparent surface) interfaces. The maps were aligned based on corresponding subunits and both densities are shown at σ=0.11. C) Comparison of the β/αD ivermectin density and model aligned with the ivermectin density and model from homomeric full-length α1GlyR (pink, PDB 6VM3) and GluCl (green, PDB 3RIA). The label refers to βI333, αA312 and G281 of αβGlyR-Gly-Ivm, homomeric α1GlyR and GluCl respectively. Alignment was done using the TM helices of the subunits forming the primary and complementary interface.

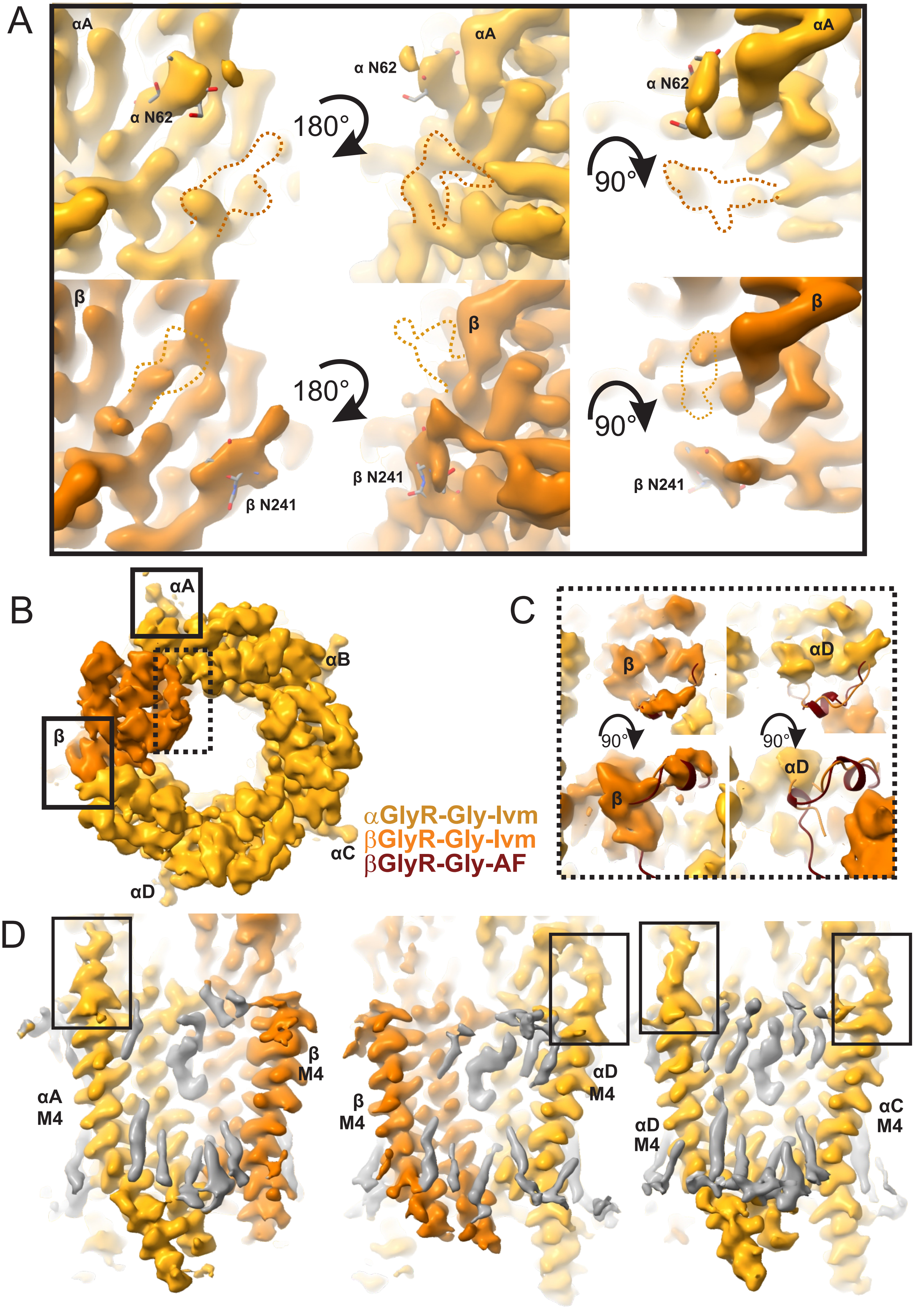

A
αA
α N62
180°
90°
β
β N241
B
C
αB
αC
αD
αGlyR-Gly-Ivm
βGlyR-Gly-Ivm
βGlyR-Gly-AF
D
αA
M4
β
M4
αD
M4
αC
M4

Supplementary Figure 8: Subunit-specific features of αβGlyR. Images are from αβGlyR-Gly-Ivm with α subunits colored in yellow and β in orange. A) Comparison of subunit-specific glycan densities at αA N62 and β N241 (All shown at σ=0.09). The glycans are shown at multiple angles and dotted lines show the outline of β N241on αA and vice-versa. B) A top-down view of the channel showing clear glycan density for each subunit, with αA N62 and β N241 outlined in solid boxes. The dotted box shows an N-terminal density associated with βGlyR. C) The βGlyR N-terminal density shown at different angles (σ=0.09). Though the density is not well defined it is consistent with the αβGlyR-Gly-Ivm model (orange) and AlphaFold βGlyR predicted model (red). D) Comparison of map density at the TMD interface between αA/β, β/D and αD/αC (σ=0.1). The densities corresponding the αGlyR M4 helices extend above the membrane representing the ten additional C-terminal residues in αGlyR compared to βGlyR. There are also differences in the lipid densities, shown in gray, at each subunit interface.

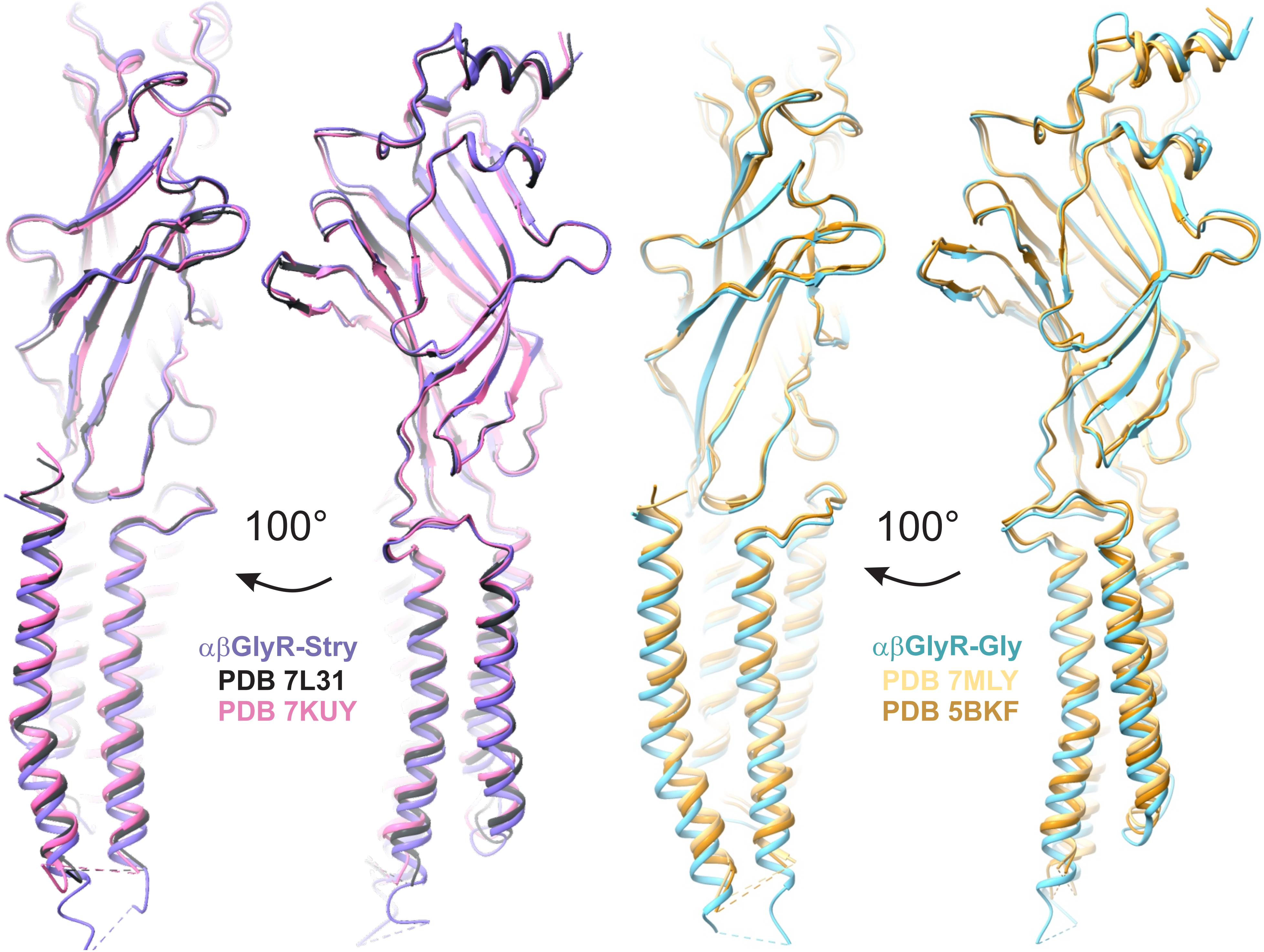
100°
αβGlyR-Stry
PDB 7L31
PDB 7KUY
100°
αβGlyR-Gly
PDB 7MLY
PDB 5BKF

Supplementary Figure 9: Comparison of published αβGlyR structures. A) The αA subunit from αβGlyR-Stry is compared to the corresponding subunit in two αβGlyR strychnine-bound structures published in Yu et al. (Strychnine-bound State 1: PDB 7L31, Strychnine-bound State 2: PDB 7KUY)[11] B) The αA subunit from the αβGlyR-Gly is compared to the corresponding subunit in previously published h-$\alpha_2\beta$GlyR glycine-bound desensitized structures in Yu et al. (Glycine bound, desensitized state: PDB 5BKF)[11] and porcine $\alpha_1\beta$GlyR Zhu et al. (Native glycine receptor: PDB 7MLY)[12].

Supplementary Table 1: **Sequence of zebrafish α_1GlyR cloned into pcDNA3.1(+)-P2A-eGFP**

using HindIII/XhoI cloning sites for expression in HEK 293T cells.

```
MFALGIYLWETIVFFSLAASQQAAARKAASPMPPSEFLDKLMGKVSGYDARIRPNFKGPPVNVTCNIFINSFGSIAE
TTMDYRVNIFLRQQWNDPRLAYSEYPDDSLDLDPSMLDSIWKPDLFFANEKGANFHEVTTDNKLLRISKNGNVLYSI
RITLVLACPMDLKNFPMDVQTCIMQLESFGYTMNDLIFEWDEKGAVQVADGLTLPQFILKEEKDLRYCTKHYNTGKF
TCIEARFHLERQMGYYLIQMYIPSLLIVILSWVSFWINMDAAPARVGLGITTVLTMTTQSSGSRASLPKVSYVKAID
IWMAVCLLFVFSALLEYAAVNFIARQHKELLRFQRRRRHLKEDEAGDGRFSFAAYGMGPACLQAKDGMAIKGNNNNA
PTSTNPPEKTVEEMRKLFISRAKRIDTVSRVAFPLVFLIFNIFYWITYKIIRSEDIHKQ
```

Supplementary Table 2: Sequence of zebrafish β_BGlyR cloned into pICherryNeo using XhoI/XbaI cloning sites for expression in HEK 293T cells.

```
MKALKVIFMLLIICLWMEGGFTKEKSAKKGKKKGKQVYCPSQLSSEDLARVPANSTSNILNKLLITYDPRIRPNFKG
IPVEDRVNIFINSFGSIQETTMDYRVNIFLRQRWNDPRLRLPQDFKSDSLTVDPKMFKCLWKPDLFFANEKSANFHD
VTQENILLFIFRNGDVLISMRLSVTLSCPLDLTLFPMDTQRCKMQLESFGYTTDDLQFMWQSGDPVQMDEIALPQFD
IKQEDIEYGNCTKYYAGTGYYTCVEVIFTLRRQVGFYMMGVYAPTLLIVVLSWLSFWINPDASAARVPLGILSVLSL
SSECTSLASELPKVSYVKAIDIWLIACLLFGFASLVEYAVVQVMLNSPKLLEAERAKIATKEKAEGKTPAKNTINGM
GSTPIHVSTLQVTETRCKKVCTSKSDLRTNDFSIVGSLPRDFELSNFDCYGKPIEVGSAFSKSQAKNNKKPPPPKPV
IPSAAKRIDLYARALFPFSFLFFNVIYWSVYL
```

Supplementary Table 3: Codon optimized zebrafish αGlyR and βGlyR sequences cloned into pFastBac-Dual using SpeI/NotI and XhoI/SphI cloning sites respectively. αGlyR is under the polyhedrin promoter and βGlyR under the p10 promoter. Note the βGlyR sequence is the reverse complement of the coding sequence as the polyhedrin and p10 promoters are in opposite directions.

__αGlyR codon optimized__

```
ACTAGTATGTTCGCCCTGGGTATCTACCTGTGGGAAACCATCGTGTTCTTCTCCCTGGCTGCTAGCCAGCAGGCTGC
TGCTCGCAAGGCCGCTTCCCCTATGCCTCCCAGCGAATTCCTGGACAAGCTGATGGGCAAGGTGTCCGGCTACGACG
CTCGCATCCGTCCCAACTTCAAGGGTCCACCTGTGAACGTCACTTGCAACATCTTCATCAACTCTTTCGGCTCAATC
GCCGAGACTACCATGGACTACAGGGTGAACATCTTCCTGAGACAGCAGTGGAACGACCCACGTCTGGCTTACTCTGA
ATACCCTGACGACTCACTGGACCTGGACCCCTCTATGCTGGACTCAATCTGGAAGCCAGACCTGTTCTTCGCCAACG
AGAAGGGCGCTAACTTCCACGAAGTGACCACTGACAACAAGCTGCTGAGGATCTCCAAGAACGGAAACGTGCTGTAC
AGCATCAGAATCACCCTGGTCCTGGCCTGCCCTATGGACCTGAAGAACTTCCCCATGGACGTCCAGACCTGCATCAT
GCAGCTGGAGTCCTTCGGTTACACTATGAACGACCTGATCTTCGAGTGGGACGAAAAGGGTGCTGTGCAGGTGGCTG
ACGGACTGACCCTGCCTCAGTTCATCCTGAAGGAGGAAAAGGACCTGCGCTACTGCACTAAGCACTACAACACCGGA
AAGTTCACTTGCATCGAGGCTCGCTTCCACCTGGAACGTCAGATGGGTTACTACCTGATCCAGATGTACATCCCCAG
CCTGCTGATCGTGATCCTGTCCTGGGTCAGCTTCTGGATCAACATGGACGCTGCTCCAGCTAGGGTGGGTCTGGGCA
TCACCACTGTCCTGACTATGACCACTCAGTCCAGCGGCTCTAGAGCTTCACTGCCCAAGGTGTCCTACGTCAAGGCC
ATCGACATCTGGATGGCTGTGTGCCTGCTGTTCGTCTTCAGCGCCCTGCTGGAGTACGCCGCTGTGAACTTCATCGC
TCGCCAGCACAAGGAACTGCTGCGTTTCCAGCGCCGTAGGAGACACCTGAAGGAGGACGAAGCTGGAGACGGAAGGT
TCTCTTTCGCCGCTTACGGCATGGGACCAGCCTGCCTGCAGGCTAAGGACGGAATGGCCATCAAGGGTAACAACAAC
AACGCTCCTACCTCAACTAACCCTCCTGAGAAGACCGTGGAGGAAATGCGCAAGCTGTTCATCTCTAGGGCCAAGAG
AATCGACACTGTGTCACGTGTCGCTTTCCCTCTGGTCTTCCTGATCTTCAACATCTTCTACTGGATCACCTACAAGA
TCATCCGCTCCGAAGACATCCACAAGCAGCTGGTTCCGCGTGGTAGTCATCACCATCACCATCACCATCACTGAGCG
GCCGC
```

__βGlyR codon optimized__

```
GCATGCTTAAGCAGGGGCCACCTGGGAAGTCTCGGTGCTGCCACCTCCAGAGCCACCTCCGCTGCCACCTCCAGCTG
GAGCCACTTGGGAAGTTTCGGTTTAGTGGTGGTGGTGGTGATGATGGTGCAGAGCTTAGGCGGGAGCCACCTGTGAA
GTTTCGGTGCCCTGGAAGTACAGGTTCTCCAGGTAGACAGACCAGTAGATCACGTTGAAGAACAGGAAGCTGAAAGG
GAACAGGGCACGAGCGTACAGGTCGATGCGCTTGGCAGCGGATGGGATGACGGGCTTAGGTGGGGGAGGCTTCTTGT
TGTTCTTGGCCTGGCTCTTGGAGAAAGCAGATCCCACTTCGATTGGCTTGCCGTAGCAGTCGAAGTTGCTCAGCTCG
AAGTCCCTGGGCAGGGATCCGACGATTGAGAAGTCGTTAGTACGCAGGTCTGACTTAGAGGTGCACACCTTCTTGCA
GCGAGTCTCGGTGACCTGCAGAGTGGACACGTGGATAGGGGTTGATCCCATGCCGTTGATAGTGTTCTTGGCTGGGG
TCTTTCCTTCAGCCTTCTCCTTAGTGGCGATCTTAGCCCTTTCGGCCTCCAGCAGCTTTGGGGAGTTCAGCATGACC
TGGACCACGGCGTATTCCACCAGTGAAGCGAAGCCGAACAGCAGGCAGGCGATCAGCCAGATGTCGATAGCCTTGAC
GTAAGACACCTTGGGCAGTTCGCTAGCCAGGGAGGTGCACTCTGAAGACAGTGACAGGACAGACAGGATTCCCAGGG
GCACACGAGCAGCAGAAGCGTCAGGGTTGATCCAGAAGCTCAGCCAGGACAGGACCACGATCAGCAGAGTGGGAGCG
TAGACACCCATCATGTAGAATCCCACTTGACGGCGCAGGGTGAAGATGACCTCCACGCAAGTGTAGTAACCGGTTCC
GGCGTAGTACTTGGTGCAGTTACCGTATTCGATGTCCTCCTGCTTGATGTCGAACTGTGGCAGAGCGATCTCGTCCA
TCTGCACAGGGTCGCCAGATTGCCACATGAACTGCAGGTCGTCAGTGGTGTAACCGAATGATTCCAGCTGCATCTTG
CAGCGCTGAGTGTCCATTGGGAACAGGGTCAGGTCCAGGGGGCAAGACAGAGTGACGCTCAGTCTCATGGAGATCAG
CACGTCACCGTTCCTGAAGATGAACAGCAGGATGTTTTCCTGGGTGACGTCGTGGAAGTTGGCGGACTTCTCGTTAG
CGAAGAACAGGTCAGGCTTCCACAGGCACTTGAACATCTTTGGGTCCACGGTCAGGCTGTCGGACTTGAAGTCCTGA
GGCAGTCTCAGCCTTGGGTCGTTCCAACGCTGGCGCAGGAAGATGTTGACGCGGTAGTCCATAGTGGTTTCCTGGAT
TGAACCGAAAGAGTTGATGAAGATGTTGACTCTGTCCTCCACAGGGATTCCCTTGAAGTTGGGTCTGATCCTAGGGT
CGTAAGTGATCAGCAGCTTGTTCAGGATGTTTGAGGTAGAGTTGGCGGGGACACGAGCCAGGTCCTCAGAAGACAGC
TGAGAAGGGCAGTACACCTGCTTGCCCTTCTTCTTGCCCTTCTTAGCGCTCTTTTCACCCTGGAAGTACAGGTTTTC
CTTCTCGAACTGTGGATGGCTCCAGCTGCCACCTCCTGAGCCACCTCCAGAGCCACCTCCCTTTTCAAATTGTGGGT
GTGACCAAGAGCCACCTCCGCTGCCACCTCCGGAGCCACCTCCCTTTTCGAACTGTGGGTGGCTCCAGGAGCCACCT
```

CCTGAGCCACCTCCAGAGCCACCTCCCTTCTCGAACTGAGGGTGAGACCACTTCTTGGCGCTCTTTTCCTTGGTGAA
GCCACCCTCCATCCACAGGCAGATGATCAGCAGCATGAAGATGACCTTCAGAGCCTTCATCTCGAG

Supplementary Table 4: Codon optimized Domain-E of rat gephyrin cloned into pFastBac1 using BamHI/EcoRI cloning sites.

Domain-E of Gephryin

```
GGATCCATGGATTACAAGGATGACGACGACAAAAGCAGCAACAACAACAACAACAACAACAACAACAACCTGGGCGA
AAACCTCTACTTCCAAGGTATCTCTCACCGTATGTCACCCTTCCCACTGACCTCCATGGACAAGGCTTTCATCACCG
TGCTGGAGATGACTCCTGTCCTGGGCACCGAAATCATCAACTACAGGGACGGAATGGGTAGAGTGCTGGCTCAGGAC
GTCTACGCCAAGGACAACCTGCCTCCCTTCCCAGCTTCTGTGAAGGACGGATACGCCGTCCGTGCTGCTGACGGACC
TGGAGACAGGTTCATCATCGGCGAGTCACAGGCTGGAGAACAGCCTACCCAGACTGTGATGCCCGGCCAGGTCATGC
GTGTGACTACCGGTGCCCCTATCCCTTGCGGAGCTGACGCTGTGGTCCAGGTCGAGGACACTGAACTGATCAGGGAG
TCCGACGACGGCACCGAGGAACTGGAAGTGCGCATCCTGGTCCAGGCTCGTCCAGGCCAGGACATCAGGCCTATCGG
ACACGACATCAAGAGGGGTGAGTGCGTGCTGGCTAAGGGAACTCACATGGGTCCCTCCGAAATCGGCCTGCTGGCCA
CCGTGGGAGTCACTGAGGTGGAAGTCAACAAGTTCCCAGTGGTCGCTGTCATGAGCACCGGCAACGAGCTGCTGAAC
CCCGAAGACGACCTGCTGCCAGGAAAGATCCGCGACTCTAACCGTTCAACCCTGCTGGCCACTATCCAGGAGCACGG
TTACCCTACTATCAACCTGGGTATCGTGGGCGACAACCCCGACGACCTGCTGAACGCTCTGAACGAGGGCATCTCCA
GAGCCGACGTGATCATCACCTCCGGTGGCGTCAGCATGGGAGAAAAGGACTACCTGAAGCAGGTGCTGGACATCGAC
CTGCACGCTCAGATCCACTTCGGTAGAGTCTTCATGAAGCCAGGCCTGCCTACCACTTTCGCCACTCTGGACATCGA
CGGAGTGCGCAAGATCATCTTCGCTCTGCCCGGTAACCCAGTCAGCGCCGTGGTCACTTGCAACCTGTTCGTGGTCC
CCGCTCTGCGCAAGATGCAGGGTATCCTGGACCCAAGACCTACCATCATCAAGGCCCGCCTGTCTTGCGACGTGAAG
CTGGACCCCCGCCCAGAGTACCACCGTTGCATCCTGACTTGGCACCACCAGGAACCTCTGCCCTGGGCTCAGTCAAC
CGGAAACCAGATGTCCAGCAGGCTGATGTCCATGAGAAGCGCCAACGGTCTGCTGATGCTGCCACCTAAGACCGAAC
AGTACGTTGAGTTGCATAAAGGTGAAGTCGTTGATGTGATGGTCATTGGTCGTTTGTAAGAGTTCGAATTC
```

Supplementary Table 5: Cryo-EM data collection, refinement and validation statistics.

Sample	αβGlyR-Stry	αβGlyR-Gly	αβGlyR-Gly-Ivm
PDBid	7TU9	7TVI	8FE1
EMDB id	26130	26141	29019
**Data Collection and processing**			
Microscope and location	Titan Krios (NYSBC)	Titan Krios (CWRU)	Titan Krios (CWRU)
Magnification	105000	81000	10500
Voltage	300	300	300
Data collection mode	counted	super-resolution (with and without CDS)	counted
Camera	K3	K3	K3
Physical pixel size	0.825 Å/pixel	1.1 Å/pixel	0.84 Å/pixel
Defocus range (uM)	−0.8 to −1.8	−0.8 to −1.8	−0.8 to −1.8
Number of movies	14,270	5808 (non-CDS)/6059 (CDS)	10326
Dose per frame	1.2 e-/Å2	1.5(non-CDS)/ 1.2 (CDS) e-/Å2	1.2 e-/Å2
Number of frames/movie	50	40 (non-CDS)/50 (CDS)	50
Initial particle number	3,012,151	3,274,091	4,231,907
Final particle number	84,437	99,183	204,512
Symmetry	C1	C1	C1
Resolution (unmasked, Å)	3.66 Å	3.80 Å	3.57 Å
Resolution (masked, Å)	2.97 Å	3.21 Å	2.98 Å
Map resolution range *	2-8	2-8	2-8
Map sharpening B-factor (Å^2)	None	None	None
**Refinement**			
Initial model used	AlphaFold	AlphaFold	AlphaFold
Composition	4alpha:1beta	4alpha:1beta	4alpha:1beta
Protein residues	1709	1687	1771
Non Hydrogen atoms	14315	13746	15434
Glycan (NAG) (molecule)	6	6	6
Glycine (molecule)	0	5	5
Strychnine (molecule)	5	0	0
PIO	10	0	0
D10	0	0	1
PLM	0	0	28
PX4	10	0	
Bonds (RMSD)			
Length (Å) (# > 4σ)	0.004(12)	0.004(0)	0.001(11)
Angles (°) (# > 4σ)	0.661(3)	0.816(8)	1.060(13)
Ramachandran plot (%)			
Outliers	0	0	0
Allowed	2.25	2.59	1.37
Favored	97.75	97.41	98.63
Rotamer outliers (%)	0	0	0
Molprobity score	1.15	1.38	1.26
Molprobity clashscore	2.99	5.16	4.89
* Local resolution range	2-8 Å	2-8 Å	2-8 Å

Supplementary Table 6: Radial Displacement and Angular Separation Between Subunit Domains.

Dist (Å) Angle(°)	αA	αB	αC	αD	β	αA/β	αB/αA	αC/αB	αD/αC	β/αD
Stry ECD	23.9	24.0	24.0	24.0	23.9	72.1	71.8	71.9	71.9	72.3
Gly ECD	24.0	24.0	24.0	23.9	24.0	72.0	72.0	71.9	71.7	72.4
Gly-Ivm ECD	23.6	23.8	23.8	23.7	23.9	71.9	72.1	71.6	71.9	72.5
Stry TMD	17.5	17.4	17.4	17.5	17.4	72.5	71.9	72.0	72.2	71.5
Gly TMD	18.9	18.7	18.8	18.7	18.7	72.0	72.0	72.2	71.6	72.2
Gly-Ivm TMD	18.6	18.7	18.7	18.6	18.7	71.9	72.2	72.0	71.9	72.1

Supplementary Table 7: PCA-Defined Tilt and Rotation Angles. Angles are defined as differences in PCA vectors going from the first listed conformation to the second. The tilt angle is defined as the difference between the PCA vectors mostly aligned with the z-direction. Tilt direction is relative to a line extending towards the pentameric axis, positive being rotated counterclockwise. The rotation angle is defined as the difference between the largest PCA within the xy-plane. Positive values are rotated counterclockwise. Though the tilt direction differs between subunits, the tilt is small enough that the net effect is minimal.

	Tilt Magnitude (°)	Tilt Direction (°)	Rotational Magnitude (°)
Stry/Gly ECD αA	5.6	12.2	7.0
Stry/Gly ECD αB	3.3	37.4	7.1
Stry/Gly ECD αC	1.4	-15.9	6.5
Stry/Gly ECD αD	3.9	-34.5	6.1
Stry/Gly ECD β	5.6	-19.0	7.0
Stry/Gly TMD αA	3.8	-113.5	-10.4
Stry/Gly TMD αB	4.1	-161.3	-11.5
Stry/Gly TMD αC	7.0	-147.6	-11.7
Stry/Gly TMD αD	7.9	-130.7	-11.1
Stry/Gly TMD β	6.2	-118.6	-10.9
Ivm/Gly ECD αA	0.3	98.2	-1.4
Ivm/Gly ECD αB	1.0	128.4	-1.3
Ivm/Gly ECD αC	0.9	138.1	-0.5
Ivm/Gly ECD αD	0.7	97.4	-1.2
Ivm/Gly ECD β	0.8	-102.6	-0.9
Ivm/Gly TMD αA	2.9	-145.9	1.0
Ivm/Gly TMD αB	2.4	-117.4	1.0
Ivm/Gly TMD αC	1.1	-104.5	1.7
Ivm/Gly TMD αD	0.9	-107.5	1.4
Ivm/Gly TMD β	1.4	-127.0	1.5

Supplementary Movie 1: Comparison of the αβGlyR-Gly-Ivm map to a previously published cryo-EM map of homomeric α1GlyR in the presence glycine and ivermectin (PDB 6VM3). The heteromeric channel is shown with α subunits in yellow, β in orange and non-protein densities in black. The entire map of homomeric α1GlyR is shown in grey. Sigma levels are indicated as changed in the movie. The movie shows sequentially, subunit-specific glycosylation of α1 and β subunits, the α1GlyR extended C-terminal region and differential ivermectin binding at the homomeric α/α interface and β/α interface.
