# Supplementary material for: Conformational transitions and allosteric modulation in a heteromeric glycine receptor
Source: Nat Commun. 2023 Mar 13;14:1363. doi: 10.1038/s41467-023-37106-7 (PMC10011588; doi:10.1038/s41467-023-37106-7)
Supplement: Supplementary file 3 — Description of Additional Supplementary Files [file 41467_2023_37106_MOESM3_ESM.pdf]

Description of Additional Supplementary Files

Supplementary Movie 1: Comparison of the αβGlyR-Gly-Ivm map to a previously published cryo-EM map of homomeric α1GlyR in the presence glycine and ivermectin (EMD-21237). The heteromeric channel is shown with α subunits in yellow, β in orange and non-protein densities in black. The entire map of homomeric α1GlyR is shown in grey. Sigma levels are indicated as changed in the movie. The movie shows sequentially, subunit-specific glycosylation of α1 and β subunits, the α1GlyR extended C-terminal region and differential ivermectin binding at the homomeric α/α interface and β/α interface.
