# Supplementary material for: Conformational transitions and allosteric modulation in a heteromeric glycine receptor
Source: Nat Commun. 2023 Mar 13;14:1363. doi: 10.1038/s41467-023-37106-7 (PMC10011588; doi:10.1038/s41467-023-37106-7)
Supplement: Supplementary file 5 — Reporting Summary [file 41467_2023_37106_MOESM5_ESM.pdf]

nature portfolio

Corresponding author(s): Sudha Chakrapani

Last updated by author(s): Feb 13, 2023

Reporting Summary

Nature Portfolio wishes to improve the reproducibility of the work that we publish. This form provides structure for consistency and transparency in reporting. For further information on Nature Portfolio policies, see our Editorial Policies and the Editorial Policy Checklist.

Statistics

For all statistical analyses, confirm that the following items are present in the figure legend, table legend, main text, or Methods section.

n/a	Confirmed	
☐	☒	The exact sample size (*n*) for each experimental group/condition, given as a discrete number and unit of measurement
☐	☒	A statement on whether measurements were taken from distinct samples or whether the same sample was measured repeatedly
☒	☐	The statistical test(s) used AND whether they are one- or two-sided<br>*Only common tests should be described solely by name; describe more complex techniques in the Methods section.*
☒	☐	A description of all covariates tested
☒	☐	A description of any assumptions or corrections, such as tests of normality and adjustment for multiple comparisons
☒	☐	A full description of the statistical parameters including central tendency (e.g. means) or other basic estimates (e.g. regression coefficient) AND variation (e.g. standard deviation) or associated estimates of uncertainty (e.g. confidence intervals)
☒	☐	For null hypothesis testing, the test statistic (e.g. *F*, *t*, *r*) with confidence intervals, effect sizes, degrees of freedom and *P* value noted<br>*Give P values as exact values whenever suitable.*
☒	☐	For Bayesian analysis, information on the choice of priors and Markov chain Monte Carlo settings
☒	☐	For hierarchical and complex designs, identification of the appropriate level for tests and full reporting of outcomes
☒	☐	Estimates of effect sizes (e.g. Cohen's *d*, Pearson's *r*), indicating how they were calculated

Our web collection on statistics for biologists contains articles on many of the points above.

Software and code

Policy information about availability of computer code

Data collection	IonFlux 16 version 5.0 (Fluxion Biosciences), Leginon v3.5 (SEMC), EPU v2.7 (ThermoFisher scientific), AcquireMP v2022 R1 (Refeyn)
Data analysis	1. Drift correction: MotionCor version 2.1.2.3<br>2. CTF estimation: GCTF version 1.06, ctffind 4.1.14<br>3. 2D and 3D Reconstruction, 3D refinement, post-processing: RELION Version 3.1 and 4.0, CryoSPARC 3.3.1, pyem version 0.5<br>4. Local resolution estimation: Resmap version 1.1.4<br>5. Pore profile calculation: HOLE version 3.0, Channel annotation package 0.9.1<br>6. Model visualization: PyMOL version 2.0.4, ChimeraX 1.1<br>7. 3D volume visualization: Chimera version 1.11.2, ChimeraX 1.1<br>8. Manual model building: Coot 0.9.4.1<br>9. Structure refinement: Phenix version 1.19.2-4158<br>10. MD simulations: Gromacs 2021<br>11. Figure generation: CorelDraw version 20.1.0.708<br>12. Electrophysiology data analysis: Clampfit version 11.2<br>13. Electrophysiology traces: OriginLab Version b9.9.0.225<br>14. Electrophysiology statistical analysis: Excel 16.6<br>15. PCA analysis: MATLAB R2018b<br>16. DiscoverMP v2022 R1 (REFEYN)<br>17. Ligand Analysis: LigPlot version 4.5.3 (EMBL-EBI)

18. Binding Pose Evaluation: gnina torch version 1.6
19. Ligand RMSD Analysis: MDAnalysis version 2.3.0

For manuscripts utilizing custom algorithms or software that are central to the research but not yet described in published literature, software must be made available to editors and reviewers. We strongly encourage code deposition in a community repository (e.g. GitHub). See the Nature Portfolio guidelines for submitting code & software for further information.

Data

Policy information about availability of data

All manuscripts must include a data availability statement. This statement should provide the following information, where applicable:

- Accession codes, unique identifiers, or web links for publicly available datasets
- A description of any restrictions on data availability
- For clinical datasets or third party data, please ensure that the statement adheres to our policy

All relevant data are available from the corresponding author upon reasonable request. The cryo-EM maps have been deposited in the Electron Microscopy Data Bank (EMDB) under accession codes EMD-26130 [https://www.ebi.ac.uk/emdb/EMD-26130] (αβGlyR-Stry), EMD-26141 [https://www.ebi.ac.uk/emdb/EMD-26141] (αβGlyR-Gly) and EMD-29019 [https://www.ebi.ac.uk/emdb/EMD-29019] (αβGlyR-Gly-Ivm). Coordinates have been deposited in the RCSB Protein Data Bank (PDB) under accession codes 7TU9 [https://www.wwpdb.org/pdb?id=pdb_00007TU9] (αβGlyR-Stry), 7TVI [https://www.wwpdb.org/pdb?id=pdb_00007TVI] (αβGlyR-Gly) and 8FE1 [https://www.wwpdb.org/pdb?id=pdb_00008FE1] (αβGlyR-Gly-Ivm).

Human research participants

Policy information about studies involving human research participants and Sex and Gender in Research.

Reporting on sex and gender	n/a
Population characteristics	n/a
Recruitment	n/a
Ethics oversight	n/a

Note that full information on the approval of the study protocol must also be provided in the manuscript.

Field-specific reporting

Please select the one below that is the best fit for your research. If you are not sure, read the appropriate sections before making your selection.

☒ Life sciences ☐ Behavioural & social sciences ☐ Ecological, evolutionary & environmental sciences

For a reference copy of the document with all sections, see nature.com/documents/nr-reporting-summary-flat.pdf

Life sciences study design

All studies must disclose on these points even when the disclosure is negative.

Sample size	For electrophysiological recordings, each sample is an ensemble of currents recorded from 20 cells attached to electrodes in a single 'trap' well of an Ionflux 16 96-well plate. No sample size calculation was made and the sample sizes were considered to be sufficient based on studies that used similar methodology.
Data exclusions	None
Replication	For IonFlux experiments, each plate contained up to 16 different samples from the same set of transfected cells. The data shown encompasses multiple plates from different days to ensure reproducibility. When a strong seal, determined by the seal resistance, was formed the reported effects were always reproduced. Data in which a strong seal was not formed, or in which the seal broke was not included.
Randomization	For IonFlux experiments, cells are placed in a 96-well plate well at a high density. Cells are then automatically perfused past electrodes and up to 20 of these cells are trapped at electrodes. This is assumed to be a random selection process of cells within the plate well.
Blinding	The experimenter was not blinded to which group cells belonged during experiments as this would not reasonably impact the results.

Reporting for specific materials, systems and methods

We require information from authors about some types of materials, experimental systems and methods used in many studies. Here, indicate whether each material, system or method listed is relevant to your study. If you are not sure if a list item applies to your research, read the appropriate section before selecting a response.

Materials & experimental systems

n/a	Involved in the study
☐	☒ Antibodies
☐	☒ Eukaryotic cell lines
☒	☐ Palaeontology and archaeology
☒	☐ Animals and other organisms
☒	☐ Clinical data
☒	☐ Dual use research of concern

Methods

n/a	Involved in the study
☒	☐ ChIP-seq
☒	☐ Flow cytometry
☒	☐ MRI-based neuroimaging

Antibodies

Antibodies used	1D4 antibody (In-house), 6x His Antibody (ThermoFisher MA1-21315), Flag Antibody (MA1-91878)
Validation	1D4 antibody was not validated beyond its use for isolating heterogeneously expressed protein. 6x His Antibody and Flag Antibody were not validated beyond the manufacture's statement.

Eukaryotic cell lines

Policy information about cell lines and Sex and Gender in Research

Cell line source(s)	ExpiSf9 cells purchased from Invitrogen. HEK-293T cells purchased from ATCC
Authentication	We have only used commercially available cell lines and have not authenticated them beyond the manufacture's statement.
Mycoplasma contamination	The cell lines were not tested for mycoplasma contamination
Commonly misidentified lines (See ICLAC register)	No commonly misidentified lines were used in this study
